# The influence of family in children’s feeding difficulties: an integrative review

**DOI:** 10.3389/fped.2025.1609714

**Published:** 2025-07-09

**Authors:** Pâmela Gracielle da Fonseca, António Raposo, Nada Alqarawi, Ibrahim Alasqah, Mariana Figueiredo Pinto, Tábata Monaliza Marcelino Martins, Viviane Dias Gonçalves, Simone Cardoso Lisboa Pereira, Najla A. Albaridi, Mona N. BinMowyna, Ariana Saraiva, Nathalia Sernizon Guimarães

**Affiliations:** ^1^ Department of Nutrition, Nursing School, Universidade Federal de Minas Gerais, Belo Horizonte, Brazil; ^2^ CBIOS (Research Center for Biosciences and Health Technologies), ECTS (School of Health Sciences and Technologies), Lusófona University, Lisboa, Portugal; ^3^ Department of Psychiatric and Mental Health, and Community Health, College of Nursing, Qassim University, Buraydah, Saudi Arabia; ^4^ Department of Nutrition, Ibirité City Hall, Ibirité, Brazil; ^5^ Department of Health Science, College of Health and Rehabilitation, Princess Nourah Bint Abdulrahman University, Riyadh, Saudi Arabia; ^6^ College of Education, Shaqra University, Shaqra, Saudi Arabia; ^7^ Research in Veterinary Medicine (I-MVET), Faculty of Veterinary Medicine, Lisbon University Centre, Lusófona University, Lisboa, Portugal; ^8^ Veterinary and Animal Research Centre (CECAV), Faculty of Veterinary Medicine, Lisbon University Centre, Lusófona University, Lisboa, Portugal

**Keywords:** avoidant restrictive food intake disorder, child, family assistance, food preferences, family relations, food selection, neophobia, preschool

## Abstract

**Background:**

Feeding difficulties, such as limited appetite, selective eating, and food phobia, affect caregivers' ability to provide adequate nutrition to children. These issues impact 25%–40% of non-neurodivergent children and up 80% of neurodivergent children.

**Aim:**

This review examines how family involvement influences the improvement, worsening, or maintenance of feeding difficulties in neurodivergent and non-neurodivergent preschool and school-age children.

**Methods:**

An integrative review was conducted using Embase, PubMed, Scopus, Cochrane Library, Lilacs and grey literature (Google Scholar and Connect Papers). The review focused on randomized clinical trials (RCTs) involving parents or caregivers of children aged 2–10 years, assessing lifestyle or psychological interventions.

**Results:**

From 1,257 studies, 885 primary articles were screened. Of the 100 most recent articles on grey literature, 2 met the eligibility criteria after full-text assessment and were therefore included in the review. Thirty-six studies were reviewed in full, leading to 11 RCTs with 630 children aged 1 to 14. Interventions included behavioral education, sensory education, and cooking classes. Findings indicated increased vegetable acceptance in two studies, improved feeding difficulties scores in five, and reduced avoidant/restrictive food intake disorder (ARFID) symptoms in two studies. One study showed no significant differences between control and intervention groups.

**Conclusion:**

Family-involved interventions generally produced positive outcomes in managing feeding difficulties. However, methodological variability and the predominance of studies from high-income countries limit the generalizability of these results. Future research should focus on standardizing diagnostic criteria and developing culturally sensitive interventions.

## 1 Introduction

Feeding difficulties (FD) refer to any condition that negatively impacts the ability of parents or caregivers to provide adequate food or nutrients to a child (1). Depending on severity, FD can lead to physical, emotional, social, and developmental consequences (2). These difficulties commonly manifest as refusal of certain food textures, extreme pickiness, or difficulties with chewing, swallowing, or other aspects of the eating process. Given the broad scope of this concept, Kerzner proposed the classification of FD into: sufficient intake (limited appetite); inadequate food intake (selective eating); fear of eating (food neophobia) (3).

Food neophobia (FN) is the reluctance to eat new foods. It peaks in childhood, around two to six years of age, and tends to decrease, stabilizing in adulthood (4) Children with food neophobia tend to have a less varied diet (5), may consume less fruit and vegetables (6), fish and meats (7), eggs (8), and like to eat more ultra-processed foods involving sugary drinks and snacks (7).

It's important to note that there's a distinction between typical “picky eating” and clinically significant FD (9). Picky eating is common in young children and usually decreases with age, while feeding difficulties FD can persist, leading to nutritional deficiencies, growth problems, and psychosocial challenges (10).

The complexity of FD has gained increasing attention in recent literature. Although no universally validated diagnostic tool exists, estimates suggest a prevalence of 25%–40% among neurotypical children and up to 80% among neurodivergent children (11). In Brazil, local studies indicate a prevalence of 37% of FD in children in a capital city of the northeastern region (12) and 43% in a capital city of the southeastern region of the country (13). Globally, the prevalence of FD varies significantly, with some studies reporting rates as high as 50% in preschool children. These statistics underscore the significant impact of FD on children health and well-being, highlighting the urgent need for effective intervention strategies.

Children with cerebral palsy (CP) frequently experience feeding difficulties due to a combination of anatomical, physiological, and behavioral factors. FD in this population are often associated with oral motor dysfunction, gastroesophageal reflux, and constipation, all of which can compromise safe swallowing and adequate nutritional intake. These challenges increase the risk of malnutrition and negatively affect growth and development. As a result, these children commonly rely on liquid or semi-solid diets and face prolonged, stressful meals. Early multidisciplinary nutritional interventions are essential to improve their quality of life and nutritional status (14). Similarly, children with Down syndrome face feeding challenges linked to muscle hypotonia, craniofacial anomalies, and sensory difficulties. These factors impair oromotor functions, including chewing and swallowing, leading to selective eating behaviors. Gastrointestinal issues such as reflux and constipation are also common and may worsen food selectivity. Supportive strategies that involve speech therapy, nutritional counseling, and occupational therapy are critical to improving their feeding skills and nutritional outcomes (15).

In both cerebral palsy and Down syndrome, food selectivity requires individualized approaches that take into account the physical, emotional and social particularities of each child. Understanding the underlying causes of these difficulties is crucial to devel-oping effective interventions that promote adequate nutritional intake and improve the child's relationship with food.

Recent studies also highlight that sociodemographic factors play a significant role in feeding difficulties among typical children. Premature birth is associated with increased risk; preterm children are 3.7 times more likely to exhibit sensory changes that affect their eating behavior compared to their peers. Additionally, younger children aged 2–3 years tend to show higher prevalence rates of feeding difficulties than older ones. Sensory alterations such as discomfort with textures or smells further exacerbate these challenges during early childhood (16).

Another relevant factor is family practices during mealtime. For instance, frequent use of screens (TVs or mobile devices) during meals has been linked to more challenging eating behaviors in children. Although caregivers often perceive this strategy as helpful for increasing food intake temporarily, it may create negative associations with mealtime routines and hinder long-term development of healthy habits. Conversely, structured family meals without distractions contribute positively to minimizing FD (16).

Eating patterns are established during childhood. When the dietary repertoire is limited, it can lead to several negative consequences, including psychological issues, motor development disorders, and learning difficulties. These impacts may persist if early interventions are not implemented (7). Studies show that sharing family meals and encouraging children to eat healthy have positive impacts on eating behavior (17). Thus, the family plays a fundamental role in shaping appropriate and healthy eating behaviors in children. The study of Wolnicka (18) demonstrated that parents' eating habits have a positive influence on children's eating repertoire, creating a beneficial relationship in behavioral change.

Parental feeding practices and children's innate behaviors are both critical to the development of feeding difficulties. However, these factors operate in distinct but complementary ways. While parenting style shapes mealtime dynamics and expectations, biological predispositions influence how children respond to food, textures, and eating routines. Therefore, effective interventions must consider both environmental (family) and individual (child-related) factors (19).

Recent studies underscore the importance of early intervention and prevention strategies to address FD in children. These strategies emphasize the role of responsive feeding practices, which involve recognizing and responding to a child's hunger and satiety cues. Responsive feeding helps children develop self-regulation skills and promotes a positive relationship with food. Additionally, creating a structured mealtime environment with consistent routines and limited distractions can also contribute to healthier eating habits (20).

The impact of culture and socioeconomic status on feeding practices is also an area of increasing research interest. Cultural beliefs and traditions often influence the types of foods offered to children, as well as the methods used to encourage eating. Families with limited financial resources may face challenges in accessing nutritious foods, which can further exacerbate feeding difficulties. Interventions that are culturally sensitive and tailored to the specific needs of families from diverse backgrounds are essential for promoting healthy eating habits in all children (21, 22).

Given the high prevalence of FD and their significant impact on children's health and family dynamics, understanding the role of the family is essential. This review aims to synthesize current evidence on how family involvement influences the improvement, persistence, or worsening of feeding difficulties in both neurotypical and neurodivergent children, providing guidance for future interventions and research.

## 2 Materials and methods

This was an integrative review conducted in the databases Embase, PubMed, Scopus, Cochrane Library, and Lilacs. The descriptors were previously searched in the MeSH, Emtree, and DeCS databases. The keywords used were: “Food Preferences”, “Food Selection”, “neophobia”, “Avoidant Restrictive Food Intake Disorder”, “Food Neophobia”, “Food Fussiness”, “Fussiness, Food”, “Picky Eating”, “Eating, Picky”, “Family Relations”, “Family Relationship”, “Relationships, Family”, “Family Supports”, “Family Encouragement”, “Family Assistance”, “Parent-Child Relations”, “Parent-Offspring Interaction”, “Parent-Child Relationship”, “Mother-Child Relations”, “Relations, Mother-Child”, “Mother-Child Relationship”, “Mother-Infant Interaction”, “Mother-Infant Relations”, “Father-Child Relations”, “Child, Preschool”. A specific and sensitive search strategy for each database was developed and is presented in Supplementary Appendix 1.

This search strategy was designed to capture a broad range of studies related to family involvement and feeding difficulties in children. The combination of MeSH terms, Emtree terms, and DeCS terms ensured that all relevant articles, regardless of indexing variations, were identified. After carrying out the searches in the aforementioned databases, we carried out a new search on Google Scholar on March 16, 2025, with the aim of identifying new studies that could be included in the review.

The inclusion criteria considered were randomized clinical trials (RCTs) involving parents or caregivers of preschoolers (1–6 years) or school-age children (6–14 years) which described as outcomes the improvement, worsening, and/or maintenance of this condition following lifestyle interventions (dietary, physical activity) or psychological interventions.

The focus on RCTs ensured a high level of evidence for the effectiveness of interventions. The age range of 2–14 years was chosen to capture studies focusing on critical periods of eating behavior development.

Studies were excluded if they were not RCTs, did not involve parents or caregivers, focused on children outside the specified age range, or did not assess the impact of interventions on FD. Just studies that involved only parental participation were included because family dynamics and parental feeding practices are central determinants of children's feeding behaviors, as supported by the theoretical framework of this review.

It is important to acknowledge that this review has inherent methodological limitations. Firstly, the exclusive focus on randomized controlled trials (RCTs) may have excluded relevant evidence from qualitative studies and observational research, which could provide valuable insights into family dynamics and feeding difficulties. Furthermore, the inclusion of studies covering a broad age range (1 to 14 years) introduces heterogeneity related to developmental stages, potentially affecting the comparability of outcomes.

Another relevant limitation is the use of intervention RCTs that combine activities designed for neurodivergent children with simpler interventions aimed at improving fruit and vegetable intake in neurotypical children. These heterogeneous interventions cannot be directly compared, as they do not fully represent real-life situations but rather reflect conceptual differences in the interpretation of interventions targeting children with entirely distinct clinical and behavioral challenges.

Nevertheless, it is important to highlight that, despite the variability in the types of interventions and populations, RCTs are characterized by high scientific rigor. Each study, within its own context and methodological design, provides valuable and reliable evidence regarding the effectiveness of family-based interventions for addressing feeding difficulties.

## 3 Results

**Figure 1:**
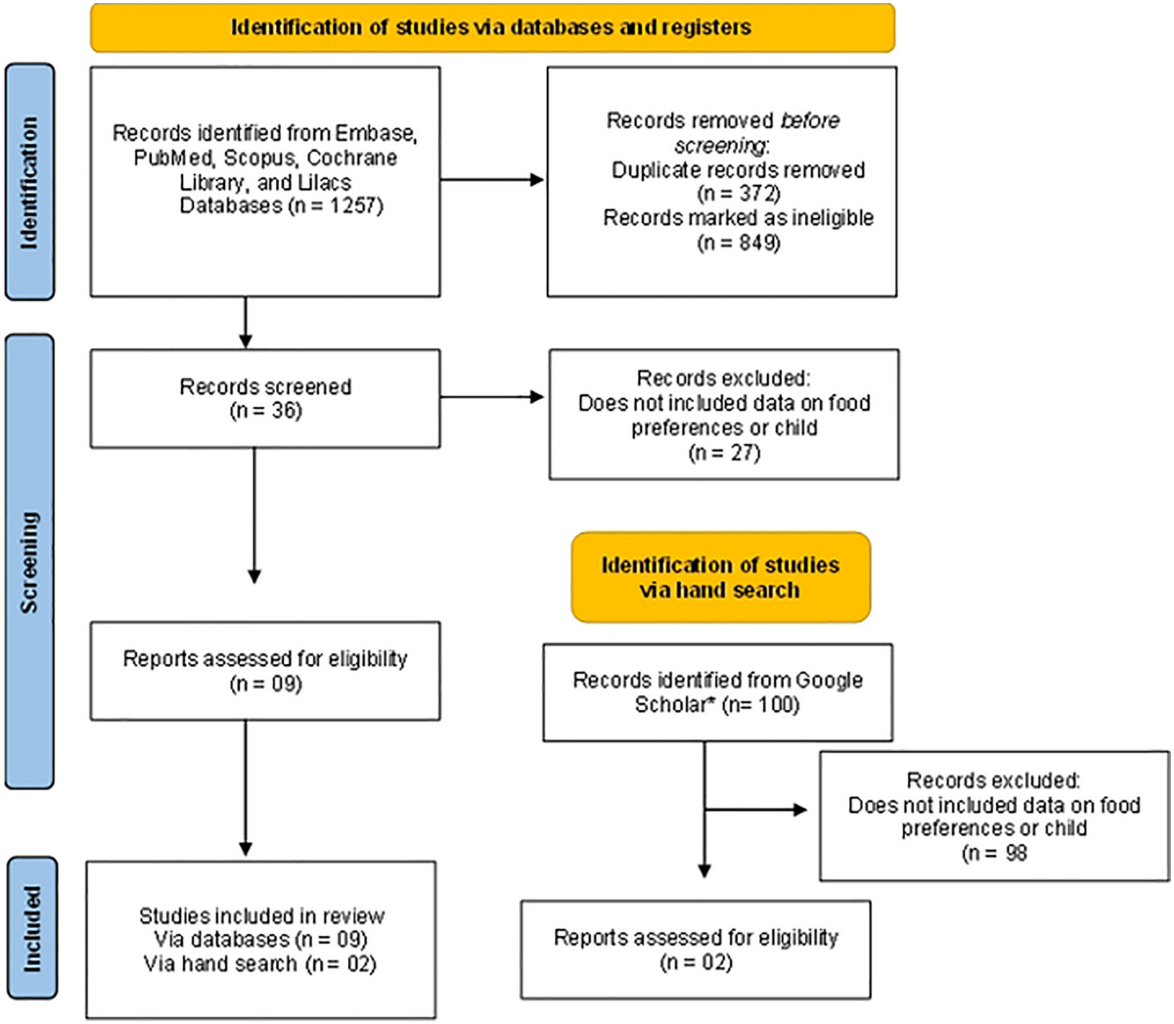
Study eligibility flowchart.

A total of 1,257 studies were initially identified in the databases. Of these, 372 were duplicates, resulting in 885 articles screened. After reviewing the titles and abstracts, 36 studies were deemed eligible and read in full. Ultimately, 11 articles were selected that met the inclusion criteria (Figure 1).

As a complementary hand search, we reviewed the 100 most recent articles retrieved from Google Scholar. Among these, 98 were excluded after title and abstract screening, and 2 met the eligibility criteria after full-text assessment and were therefore included in the review.The 11 RCTs included a total of 630 children aged 1 to 14 years. Of these, 326 were boys (51.7%), 298 were girls (47.3%) and, in one study with 6 participants (1%), gender was not specified. All selected studies were published in high-income countries between 2016 and 2021. The majority of the participants were neurodivergent children (23–29). However, three studies exclusively included children with Autism Spectrum Disorder (ASD) (30–32), Attention Deficit Hyperactivity Disorder (ADHD), and anxiety, as well as children without neurodevelopmental disorders (33). Table 1 summarizes the main characteristics of the interventions, including target populations, intervention strategies, main outcomes, and reported effectiveness. Supplementary Table S1, available in the Supplementary Material, presents the individual results of each study, with detailed information on study design, sample size, follow-up period, assessment tools, and outcome measures.

**Table 1:** Summary of interventions, target populations, outcomes, and reported effectiveness in the included studies.

Study (Author, Year)	Population	Intervention type	Main outcome	Effectiveness
Blomkvist et al., 2021 (23)	Neurodivergent (Food Neophobia)	Sensory Education (Sapere Method) + Parental Guidance	Increased vegetable intake; no change in neophobia	Moderate
Park et al., 2016 (24)	Neurodivergent (Food Neophobia)	Taste Education + Cooking Practice	Reduced food neophobia; increased willingness to try	High
Skouteris et al., 2016 (25)	Neurodivergent (Food Neophobia)	Behavioral + Parental Lifestyle Training	Reduced neophobia at 12-month follow-up	High (Sustained)
Skouw et al., 2020 (26)	Neurodivergent (Food Neophobia)	Serious Game—Sensory Exposure	Improved eating behaviors; decreased neophobia	Moderate to High
Dahlsgaard et al., 2019 (27)	Neurodivergent (ARFID)	Cognitive Behavioral—Parent Training	Reduced picky eating; sustained after 3 months	High (Sustained)
Lock et al., 2019 (28)	Neurodivergent (ARFID)	Family-Based Treatment (FBT-ARFID)	Improved ARFID symptoms (large effect)	High
Shimshoni et al., 2020 (29)	Neurodivergent (ARFID)	Parent-based Anxiety-Focused (SPACE-ARFID)	Reduced ARFID severity; improved dietary flexibility	High
Sharp et al., 2019 (30)	Children with ASD	MEAL Plan (Parent + Child Sessions)	Improved variety and reduced aversion (BAMBI)	High
Crowley et al., 2020 (31)	Children with ASD (Food Selectivity)	Behavioral—Reinforcement with Food Choice	Increased consumption of alternative foods	Moderate to High
Peterson et al., 2019 (32)	Children with ASD (Food Selectivity)	Behavioral Analytic Therapy	Increased food acceptance; improved variety	High
Thorsteinsdottir et al., 2021 (33)	Children with and without ND	Taste Education with Cooking Activities + Parental Sessions	Reduced food selectivity; improved variety in diet	High

See Supplementary Table S1 for detailed individual study characteristics and outcome data.

FD, feeding difficulties; ASD, autism spectrum disorder; ADHD, attention-deficit/hyperactivity disorder; OCD, obsessive-compulsive disorder; ARFID, avoidant/restrictive food intake disorder; FBT-ARFID, family-based treatment for ARFID; SPACE-ARFID, supportive parenting for anxious childhood emotions adapted for ARFID; MEAL Plan, managing eating aversions and limited variety plan; BAMBI, brief autism mealtime behavior inventory; SAPERE, sensory-based educational approach; RCT, randomized controlled trial; ND, neurodivergent.

The diversity in study populations, including both neurotypical and neurodivergent children, allowed for a comprehensive assessment of the impact of family involvement on feeding difficulties across different developmental profiles.

To assess FD in children, the criteria varied. One or more questionnaires were used such as the Food Neophobia Scale (FNS) (23–26), Children's Eating Behavior Questionnaire (CEBQ) (25, 27, 33) e Willingness to Try Novel Foods (WTNF) (24), Avoidant/restrictive food intake disorder (ARFID) diagnosis according Diagnostic and Statistical Manual of Mental Disorders (DSM-5) (27–29, 31) and measures of food acceptance and variety based on the consumption of specific food items reported by parents (24, 26, 32, 33).

The professional interventions consisted of weekly sessions lasting 60–120 min, involving both parents and children, except for one that included only the parents (27). Of the studies that addressed neuroatypical individuals, they all used interventions focused on behavioral education, with the goal of equipping parents with strategies to improve feeding practices and family dynamics (30, 33). Similarly, two studies with neurotypical children also adopted this strategy (29). Sensory education-focused interventions (23, 26, 31, 32) and cooking classes (24, 33) were also observed aimed at promoting the acceptance of new foods and developing practical skills. Finally, one study based its intervention on learning and lifestyle theories, focusing on education about nutrition and healthy behaviors (25). The intervention duration ranged from 3 weeks to 20 months.

Considering the different diagnostic criteria adopted, the results varied regarding the outcomes evaluated. With respect to the intake and acceptance of new foods, it was observed that the interventions were effective in increasing the acceptability of vegetables (23, 33).

Regarding the scores on questionnaires assessing FD, it was observed that most showed an improvement in the scores (23–33) except for one study that did not observe any difference between the control and intervention groups. This suggests that while many interventions are effective, not all approaches are universally successful, highlighting the importance of tailoring interventions to individual needs (23). Regarding the reduction of ARFID symptoms, it was observed that the two studies evaluating this variable reported positive results, reflecting a decrease in the severity of the condition (28, 29).

Interventions based on parental education have been shown to be effective, especially for children with ASD. The Autism MEAL Plan (30) achieved a 47.4% positive response rate, outperforming generic parenting education (5.3%). However, approaches targeting ARFID (28, 29) showed feasibility but no proven long-term effectiveness.

School and sensory interventions have shown potential in reducing food neophobia. Sensory education in schools (33) and the Sapere method (23) increased acceptance of new foods, although the overall impacts were variable.

Innovative technologies, such as digital games (26), have shown potential in reducing food resistance, but still lack validation in larger samples.

Behavioral approaches, especially Applied Behavior Analysis (ABA) (31, 32), have been effective in increasing food acceptance in children with ASD. However, their applicability to other disorders, such as ARFID, still needs to be further investigated.

## 4 Discussion

In recent studies have shown that behavioral analytic interventions are widely used to treat feeding problems in children, particularly those with autism and ARFID. These interventions often include antecedent components, such as modeling and high-probability sequences, which have been effective in increasing the acceptance of new and non-preferred foods. Additionally, multidisciplinary interventions, incorporating oral motor therapy and behavioral approaches, have been demonstrated to significantly improve food acceptance and consumption in children with feeding difficulties. These findings underscore the importance of integrating behavioral and sensory strategies in interventions, acknowledging the complex interplay between aversive behaviors and sensory sensitivities that characterize many feeding challenges (16). However, the literature lacks studies evaluating the effectiveness of these interventions in low- and middle-income countries, where challenges are more complex and multifaceted (34).

A study published in 2023 reported a prevalence of FD in young children as high as 31.4%, with an increasing trend over time. This study identified several risk factors for FD, including frequent constipation, parental anxiety, indulgent parenting style, luring to eat, forcing to eat, and allowing playing during mealtime. Conversely, protective factors such as food preparation, observing hunger and satiety signals, interacting with the child during mealtime, and providing exclusive tableware were also noted. Prospective research is needed to investigate whether these early feeding behaviors predict long-term eating habits and psychological well-being. These variables need to be carefully examined in intervention programs for promoting healthy eating behaviors and preventing eating disorders in later life (35). Furthermore, research also suggests that children with FD are more likely to experience mental health issues, such as anxiety and depression (36).

These findings highlight the importance of parental involvement in feeding interventions. Research suggests that parents who use positive education strategies, such as guidance on proper and healthy eating, achieve better results compared to those employing negative practices like coercion or rewards. This aligns with the findings of eleven studies that evaluated interventions involving parents and family members to address FD in children. After these interventions, two studies showed an increase in vegetable acceptance, seven reported improvements in FD questionnaire scores, and two observed a reduction in ARFID symptoms. Only one study did not find differences between the control and intervention groups. These results support previous research suggesting the critical role of parental involvement in feeding interventions (23–33). Moreover, early support should be provided to caregivers of children with increased FD. Professionals must encourage families to develop responsive feeding practices as part of an interdisciplinary team focused on promoting appropriate child development, improving food acceptance, decreasing parental stress, and strengthening parent-child interaction (15, 16).

The family environment plays a crucial role in the learning process, particularly in the strategies parents use to encourage their child's eating or teach them to consume specific foods. A systematic review found a statistically significant association between the use of parental pressure to feed and selective eating in children. Similarly, a study evaluating the association between parental practices and Children eating behavior demonstrated that parents who used positive education, such as guidance on proper and healthy eating, achieved better results compared to parents whose practices were considered negative, such as coercion, reward, and food restriction. In this sense, evidence shows that it is essential to guide and empower parents to promote effective changes in their Children behavior. This allows them to adopt more effective and positive approaches based on encouragement and support, rather than pressure or coercion. However, this requires ongoing support from healthcare professionals through multidisciplinary approaches. Unlike unprofessional methods, multidisciplinary teams work in an integrated and cooperative manner across various contexts, resulting in shorter intervals between consultations, increased effectiveness of assessments, and better understanding of problems (37–43).

On the other hand, a critical analysis of the studies identified some limitations. First, there is variability in the methods of the included studies, including the diagnosis of FD, proposed interventions, and evaluated outcomes. This disparity complicates the comparison between studies and the generalization of results. Second, the predominance of studies conducted in high-income countries limits the extrapolation of results to other countries with significantly different socioeconomic and cultural contexts. In low- and middle-income countries, families often face additional challenges such as food insecurity, limited access to health and education services, and different cultural practices and perceptions about food. These factors may influence the approach to FD, making strategies effective in high-income countries potentially inadequate or ineffective in lower-income contexts. Finally, the limited duration of professional interventions raises concerns about the sustainability of the achieved results. Although studies identified immediate improvements in Children eating behavior, there was no long-term follow-up to verify the persistence of these gains. Thus, there is a risk of regression to previous eating patterns (44).

As the context of feeding difficulties varies significantly between high-income countries (HICs) and low- and middle-income countries (LMICs) such as Brazil, it is crucial to highlight the distinct challenges faced by families in these settings. In Brazil, recent studies indicate that food insecurity significantly impacts dietary practices among children. For instance, a study conducted in Maceió found that approximately 76% of children lived in food-insecure households, which negatively affected their nutritional status and overall health outcomes (45). This statistic underscores the correlation between socioeconomic vulnerabilities and inadequate feeding practices, often compounded by cultural beliefs and limited access to education that influence parenting and nutrition decisions (46). The nutritional landscape marked by a transition toward ultra-processed foods poses further risks, with childhood obesity rates climbing alongside persistent malnutrition issues, revealing the dual burden that affects many Brazilian households. One systematic review and meta-analysis reported that contextual factors, including the prevalence of childhood obesity, were higher in settings with greater access to ultra-processed foods, suggesting a complex relationship between food availability and dietary quality (47). Moreover, the interrelation of maternal mental health and food security in the Brazilian context presents additional layers of complexity in understanding feeding difficulties. Studies have demonstrated that maternal distress is correlated with higher risks of food insecurity during pregnancy, exacerbating existing vulnerabilities faced by families (46). Questions surrounding the sustainability and accessibility of effective pediatric nutritional interventions remain critical, as existing policies aimed at bolstering early childhood nutrition often overlook these multifaceted issues rooted in socioeconomic status and mental health. Interventions need to be culturally tailored and sensitive to the unique barriers encountered by families in Brazil (48). Therefore, it is imperative to develop and assess family-centered interventions that consider not only behavioral challenges but also the broader structural factors affecting food security and nutrition among children in LMICs.

In terms of assessment tools, a scoping review highlighted the variety of instruments available for evaluating feeding problems in children, including the Montreal Children Hospital Feeding Scale and the Children Eating Behavior Questionnaire. These tools are essential for identifying and addressing FD early on (49).

FD are not limited to healthy children; they are also prevalent in children with specific medical conditions. For instance, children with esophageal atresia often experience significant feeding challenges that affect their growth and nutrient intake, as well as those with cerebral palsy who often face challenges such as loss of food through the mouth, choking and vomiting, associated with stature-weight deficits and the need for interventions such as gastrostomy. Children with Down syndrome, on the other hand, have motor and cognitive deficits that impact chewing and swallowing, requiring dietary adaptations and specialized support to avoid nutritional complications. In all these cases, personalized interventions—including speech therapy, gastroenterological and nutritional guidance—are essential to optimize calorie intake, prevent malnutrition and improve quality of life (14, 15, 50).

Emerging research also focuses on the impact of the COVID-19 pandemic on children eating habits and FD. Lockdowns, school closures, and changes in family routines have disrupted mealtime environments and increased stress levels, potentially exacerbated existing FD or contributed to the development of new ones. Studies are needed to understand the long-term effects of the pandemic on children eating behaviors and to develop strategies for mitigating these effects (51–54). However, most studies did not assess the long-term impact of the pandemic. Future research should examine the long-term effects of changes in family routines and stress levels during the pandemic on children eating behaviors and FD, in addition to investigate the role of technology-based interventions in supporting families during disruptions such as the COVID-19 pandemic.

One of the main difficulties observed in the randomized controlled trials (RCTs) analyzed in this review was the high rate of participant resistance throughout the studies, often due to health-related issues. Additionally, the lack of standardization in the instruments and outcome measures used across studies contributes to significant heterogeneity in the results, complicating data comparison and interpretation. Another critical challenge relates to the heterogeneity of the populations and the intervention designs themselves.

Children with sensory processing disorders, neurodevelopmental conditions such as ASD, ADHD, or OCD, and those with organic disorders like food allergies or intolerances present fundamentally different feeding challenges. Consequently, interventions tailored for neurodivergent children—often intensive and behavioral—are conceptually and practically distinct from simpler interventions aimed at improving dietary variety in neurotypical children. Furthermore, the diagnostic criteria for Avoidant/Restrictive Food Intake Disorder (ARFID) must be rigorously applied, as this diagnosis pertains to children with severe and complex feeding problems, substantially different from those exhibiting typical picky eating. This variability across studies poses substantial challenges to comparing the effectiveness of interventions and drawing generalized conclusions.

For interventions to have a real and lasting impact, it is essential that they are multifaceted, incorporating strategies such as parental training, sensory-based education, behavioral approaches, and environmental modifications. These comprehensive approaches have demonstrated greater effectiveness in reducing food selectivity, as they simultaneously address the multiple factors influencing children's eating behaviors. However, the variability in intervention formats further highlights the need for careful interpretation of results when comparing studies with different targets, populations, and methodologies.

It is also important to highlight the notable absence of longitudinal studies within the body of RCTs analyzed. Most of the reviewed trials adopt relatively short follow-up periods, limiting the assessment of long-term effects of the interventions. Studies with extended follow-up can offer more robust insights into the persistence of food selectivity and the factors influencing its evolution over time. Continued follow-up is essential not only for understanding the developmental trajectories of FD but also for capturing the contextual and familial factors that contribute to their maintenance or resolution.

The findings of this review have clear implications for both clinical practice and public health policy.

Healthcare professionals should integrate family-centered strategies into their routine care for children with FD, assessing family dynamics, mealtime routines, and parental feeding practices. Interventions that empower parents through training in responsive feeding strategies, positive discipline, and environmental structuring are critical to improving children's eating behaviors. From a public policy perspective, it is essential to develop and implement programs that support parental education, provide accessible nutritional guidance, and ensure families, especially those in socioeconomically vulnerable contexts, have access to healthy foods and resources that promote positive feeding environments.

Technology-based interventions represent a promising complement to traditional strategies. Mobile applications offering tools for meal planning, behavioral tracking, and interactive guidance can support parents in managing FD more effectively. Telehealth services provide expanded access to multidisciplinary care, particularly for families in remote or underserved regions. Additionally, online support communities and educational platforms foster peer support and shared learning, helping parents navigate challenges related to FD. Nevertheless, these technological tools must be flexible and adaptable to meet the diverse needs of both neurotypical and neurodivergent children, ensuring cultural and contextual relevance (55–57).

Addressing the gaps identified in this review is critical for advancing the field. Future research should prioritize longitudinal RCTs to assess the sustainability of intervention outcomes over time. Additionally, the development of culturally adapted and standardized assessment tools is necessary to improve the comparability of studies. There is also a need for research that differentiates intervention strategies based on specific child profiles—whether neurotypical, neurodivergent, or children with ARFID or organic conditions—ensuring that each group receives tailored and effective support.

## 5 Conclusions

This review reinforces the critical role of family-centered interventions in managing FD in both neurotypical and neurodivergent children. Evidence indicates that strategies involving parents are effective in improving children's eating behaviors by addressing the multifactorial nature of FD. However, the considerable heterogeneity of study populations, intervention designs, and outcome measures highlights the need for caution when interpreting the results and generalizing findings.

The current literature is further limited by the predominance of studies with short follow-up periods, the lack of standardized assessment tools, and insufficient differentiation between child profiles, such as neurotypical, neurodivergent, or those with ARFID or organic disorders. Addressing these gaps is crucial for the development of more effective and culturally sensitive interventions.

Future research should prioritize longitudinal RCTs, the development of culturally adapted and standardized instruments, and the design of tailored interventions according to the specific needs of distinct clinical populations. These advances will contribute to more robust evidence and enhance the quality of care and support provided to children and families facing FD.
