# Supplementary material for: The influence of family in children’s feeding difficulties: an integrative review
Source: Front Pediatr. 2025 Jul 9;13:1609714. doi: 10.3389/fped.2025.1609714 (PMC12283677; doi:10.3389/fped.2025.1609714)
Supplement: Supplementary file 1 [file Table1.docx]

| Author  **Table S1:** Description of the individual characteristics of the studies evaluated in this integrative review, 2025. | Country | Study type | Population | Age | Follow-up | Diagnosis criteria | Intervention | Outcomes | Results |
| --- | --- | --- | --- | --- | --- | --- | --- | --- | --- |
| Blomkvist *et al.*, 2021 [23] | Norway | Randomized clinical trial | Neurodivergent children (n=144; ♂=73) | 1 year | 12 weeks | 6-item version of the Child Food Neophobia Scale (CFNS) proposed by Pliner (1994) | Sensory lessons (Sapere Method) for children at school and food recommendations for parents, addressing food neophobia and repeated exposure to food. | Vegetable intake, as well as the level of post-intervention food neophobia | Higher vegetable intake in the diet + Sapere method group compared to the control group; no effect observed on food neophobia. |
| Park *et al.,* 2016 [24] | Seul | Randomized clinical trial | Neurodivergent children (n=101; ♂=29) | 7-9 years | 12 weeks | *Food Neophobia Scale* (FNS) proposed by Pliner (1994) and *Willingness to Try Novel Foods* (WTNF) | Taste education program: Education activities on the five tastes, repeated cooking practice and group activities. Frequency: 12 sessions of 100 minutes once a week. | Changes to the FNS and WTNF scores. | Significant reduction in food neophobia in the post-test, with an increase in WTNF. |
| Skouteris *et al*., 2016 [25] | Australia | Randomized clinical trial | Neurodivergent children (n=171; ♂=85) | Mean age: 2,7 years | 15 meses | *Children’s Eating Behaviour Questionnaire* (CEBQ) and *Food Neophobia Scale* (FNS) | MEND 2-4: based on theories of learning and social cognition focused on nutrition, physical activity, parenting and lifestyle behaviors. Frequency: 10 weekly workshops of 90 minutes each. | Changes in CEBQ and FNS scores. | Neophobia (FNS) was lower in the intervention group compared to the control group 12 months after the intervention. |
| Skouw *et al*., 2020 [26] | Denmark | Randomized clinical trial (pilot with results) | Neurodivergent children (n=18; ♂=9) | Mean age: 9 years | 3 weeks | Self-developed questionnaire with 3 parts: *Food Neophobia Scale* (FNS), a field to describe the fruits and vegetables presented (a measure of food vocabulary); and willingness to taste the fruits and vegetables presented (yes/no). | The Kingdom of Taste game: involves exposure to new or unappreciated foods through sensory interactions. Frequency: Families were instructed to carry out their assigned tasks at home once a week. | Changes in the questionnaire developed, as well as observed changes in eating behavior at home during the intervention period; changes in willingness to taste food, how they discussed fruits and vegetables (F&V), and changes in children's involvement with fruits and vegetables. | Qualitative and quantitative results showed a positive change in eating behavior and a decrease in food neophobia in all groups. |
| Dahlsgaard *et al*., 2019 [27] | United States | Randomized clinical trial (pilot with results) | Neurodivergent children (n=21; ♂=19) | 4-11 years | 23 weeks | ARFID diagnosis: through a clinical interview based on DSM-5 criteria, as well as information on symptom severity and functional impairment obtained from the battery of standardized parent-report questionnaires (BPFAS, CEBQ, CBCL) | Group treatment (cognitive behavioral): Seven sessions for parents, focused on training for food displays and management of problem behaviors during meals. Frequency: 7 sessions of 90 minutes. | Changes in the BPFAS, CEBQ and CBCL score. | Significant reductions in picky eating from pre- to post-treatment, with moderate to large effects maintained for three months. |
| Lock *et al*., 2019 [28] | United States | Randomized clinical trial | Neurodivergent children (n=28; ♂=14) | Mean age: 9 years | 12 weeks | Diagnosis of ARFID by clinicians specializing in eating disorders using DSM-5 criteria | FBT-ARFID: Interventions such as externalization, agnosticism, parental empowerment and a behavioral focus on changing eating behavior. Frequency: the final decision on the frequency and duration of treatment was based on agreement between therapist and parents. | *The Pica, ARFID, Rumination Disorder Interview* (PARDI), *Child Eating Attitudes Test* and *Strengths and Difficulties Questionnaire.* | When examining the score changes between the groups in the PARDI severity ratings, a large ES of 0.83 was found in favor of those who received FBT-ARFID. |
| Shimshoni *et al.*, 2020 [29] | United States | Randomized clinical trial (pilot with results) | Neurodivergent children (n=15; ♂=13) | 6–14 years | 12 weeks | Diagnosis of ARFID based on DSM-5 criteria | *Supportive Parenting for Anxious Childhood Emotions* adaptado para ARFID (SPACE-ARFID):focuses on parents' behavior, particularly their responses to the child's symptoms. Frequency: 12 meetings of 60 minutes once a week. | *Nine Item Avoidant/Restrictive Food Intake Disorder Screen* (NIAS) and dietary flexibility (eating completely new foods; eating new brands/appearances of favorite foods; eating new flavors of favorite foods; eating in new environments; flexibility in other aspects of eating). | The severity of ARFID symptoms and impairment, as well as family adaptation, were significantly reduced from pre- to post-treatment. In addition, increases in dietary flexibility were described. |
| Sharp *et al*., 2019 [30] | United States | Randomized clinical trial | Children with ASD (Autism Spectrum Disorder). (n=38; ♂=32) | Mean age: 5 years | 16 weeks | Own criteria: ≥6 total food items, ≥1 fruit or vegetable, as well as ≥1 items from the other food categories (i.e. protein, grain and dairy), and 2 or fewer food items in 1 food category (i.e. fruit, vegetable, protein, grain or dairy) | MEAL Plan: Intervention for eating difficulties in ASD, including nutritional planning and meal structuring. First four sessions for parents; last sessions with meal demonstrations between parents and children. Frequency: 10 90-minute group sessions over 12 weeks. | Scale improvements | Children in the MEAL Plan group scored significantly lower on the BAMBI compared to the control group. |
| Crowley *et al.,* 2020 [31] | United States | Randomized clinical trial | Children with ASD (Autism Spectrum Disorder). (n= 7; ♂=5) | 2-8 years | 20 months | Diagnosis of ARFID based on DSM-5 criteria | The intervention consisted of offering participants the choice between alternative foods and their change-resistant foods in different presentation conditions, reinforcing the consumption of the alternative foods through positive contingencies, while caregivers were trained to implement the protocol effectively. | The outcomes measured in the study included the consumption of alternative foods, the choice between foods during different presentation conditions, inter-observer agreement, and the consumption of foods resistant to change, allowing the effectiveness of the intervention to be assessed. | Os principais resultados mostraram que, durante a condição de escolha assimétrica, todos os participantes aumentaram significativamente o consumo de alimentos alternativos em comparação com a escolha livre, com alguns alcançando níveis elevados e estáveis, embora a resposta fosse inconsistente entre os participantes, sugerindo que fatores individuais podem influenciar os resultados. |
| Peterson *et al.,* 2019 [32] | United States | Randomized clinical trial | Children with ASD (Autism Spectrum Disorder). (n= 6) | 3-10 years | 24 weeks | Diagnosis of ARFID based on Autism Diagnostic Observation Schedule II. | Behavior-analytic intervention focused on food acceptance. Frequency: 24 weeks of 1,5-hour appointments once a week. | Percentage increase in independent acceptance of food and oral cleaning. | An Applied Behavior Analytic intervention effectively increased the variety of foods accepted by children with Autism Spectrum Disorder (ASD) who exhibited food selectivity, leading to significant improvements in food acceptance and consumption compared to a waitlist control group. The positive effects of the intervention were maintained during the follow-up period, and parents reported high satisfaction, highlighting its acceptability and feasibility in real-world settings. |
| Thorsteinsdottir *et al.,* 2021 [33] | Iceland | Randomized clinical trial | Children with and without neurodevelopmental disorders (n=81; ♂=47) | 8-12 years | 7 weeks | *Children’s Eating Behaviour Questionnaire* (CEBQ) and measures of food acceptance and variety based on the intake of certain food items reported by parents | The Taste Education: Two parental education sessions, followed by six sessions in the kitchen with food preparation activities, led by a psychologist and nutritionist. Frequency: 2 sessions of 2h and 6 sessions of 1h30min. | Changes in the CEBQ score and in the percentage of foods accepted. | The intervention was more effective than waiting in reducing food selectivity, with stable improvements over six months. Acceptance of vegetables, nuts and seeds increased, but there was no significant impact for fruit. Trends were similar for children with and without ND. |

**References:**

23. Blomkvist, E. A. M., Wills, A. K., Helland, S. H., Hillesund, E. R., & Øverby, N. C.. Effectiveness of a kindergarten-based intervention to increase vegetable intake and reduce food neophobia amongst 1-year-old children: a cluster randomised controlled trial. *Food Nutr. Res.* 2021, *65*. <https://doi.org/10.29219/fnr.v65.7679>.

24. Park, B.-K.; Cho, M.-S. Taste education reduces food neophobia and increases willingness to try novel foods in school children. *Nutr. Res. Pract.* 2016, *10*, 221–228. <https://doi.org/10.4162/nrp.2016.10.2.221>

25. Skouteris, H., Hill, B., McCabe, M., Swinburn, B., & Busija, L. A parent-based intervention to promote healthy eating and active behaviours in pre-school children: evaluation of the MEND 2-4 randomized controlled trial: Promoting healthy behaviours in pre-school children. *Pediatr. Obes.* 2016, *11*, 4–10. <https://doi.org/10.1111/ijpo.12011>

26. Skouw, S., Suldrup, A., & Olsen, A. A serious game approach to improve food behavior in families—A pilot study. *Nutrients* 2020, *12*, 1415. <https://doi.org/10.3390/nu12051415>.

27. Dahlsgaard, K.K.; Bodie, J. The (extremely) picky eaters clinic: A pilot trial of a seven-session group behavioral intervention for parents of children with avoidant/restrictive food intake disorder. *Cogn. Behav. Pract.* 2019, *26*, 492–505. <https://doi.org/10.1016/j.cbpra.2018.11.001>

28. Lock, J.; Sadeh-Sharvit, S.; L’Insalata, A. Feasibility of conducting a randomized clinical trial using family-based treatment for avoidant/restrictive food intake disorder. *Int. J. Eat. Disord.* 2019, *52*, 746–751. <https://doi.org/10.1002/eat.23077>

29. Shimshoni, Y.; Silverman, W.K.; Lebowitz, E.R. SPACE-ARFID: A pilot trial of a novel parent-based treatment for avoidant/restrictive food intake disorder. *Int. J. Eat. Disord.* 2020, *53*, 1623–1635.  <https://doi.org/10.1002/eat.23341>

30. Sharp, W. G., Burrell, T. L., Berry, R. C., Stubbs, K. H., McCracken, C. E., Gillespie, S. E., & Scahill, L. The autism Managing Eating Aversions and Limited variety Plan vs parent education: A randomized clinical trial. *J. Pediatr.* 2019, *211*, 185–192.e1. <https://doi.org/10.1016/j.jpeds.2019.03.046>.

31. Crowley JG, Peterson KM, Fisher WW, Piazza CC. Treating food selectivity as resistance to change in children with autism spectrum disorder. *J Appl Behav Anal*. 2020;53(4):2002-2023. doi:10.1002/jaba.711.

32. Peterson KM, Piazza CC, Ibañez VF, Fisher WW. Randomized controlled trial of an applied behavior analytic intervention for food selectivity in children with autism spectrum disorder. *J Appl Behav Anal*. 2019;52(4):895-917. doi:10.1002/jaba.650.

33. Thorsteinsdottir, S., Njardvik, U., Bjarnason, R., Haraldsson, H., & Olafsdottir, A. S. Taste education—A food-based intervention in a school setting, focusing on children with and without neurodevelopmental disorders and their families. A randomized controlled trial. *Appetite* 2021, *167*, 105623. https://doi.org/ 10.1016/j.appet.2021.105623.
