# Supplementary material for: The influence of family in children’s feeding difficulties: an integrative review
Source: Front Pediatr. 2025 Jul 9;13:1609714. doi: 10.3389/fped.2025.1609714 (PMC12283677; doi:10.3389/fped.2025.1609714)
Supplement: Supplementary file 2 [file Supplementaryfile1.docx]

Appendix A

Appendix A.1 – Search strategy

(((("Food Preferences"[Mesh] OR "Food Preference" OR "Preference, Food" OR "Preferences, Food" OR "Food Selection" OR "Food Selections" OR "Selection, Food" OR "Selections, Food" OR "food selectivity" OR "neophobia" OR "Avoidant Restrictive Food Intake Disorder"[Mesh] OR "ARFID" OR "Food Neophobia" OR "Food Neophobias" OR "Neophobia, Food" OR "Food Fussiness"[Mesh] OR "Food Fussinesses" OR "Fussiness, Food" OR "Picky Eating" OR "Eating, Picky") AND ("Family Relations"[Mesh] OR "Family Relation" OR "Relation, Family" OR "Relations, Family" OR "Family Relationship" OR "Family Relationships" OR "Relationship, Family" OR "Relationships, Family" OR "Family Dynamics" OR "Dynamic, Family" OR "Dynamics, Family" OR "Family Dynamic" OR "Family Support"[Mesh] OR "Family Supports" OR "Support, Family" OR "Family Encouragement" OR "Encouragement, Family" OR "Family Encouragements" OR "Family Assistance" OR "Assistance, Family" OR "Family Assistances" OR "Parent-Child Relations"[Mesh] OR "Parent Child Relations" OR "Parent-Child Relation" OR "Relation, Parent-Child" OR "Parent Child Relationship" OR "Parent Child Relationships" OR "Relationship, Parent Child" OR "Parent-Offspring Interaction" OR "Interaction, Parent-Offspring" OR "Parent Offspring Interaction" OR "Parent-Offspring Interactions" OR "Parent-Child Relationship" OR "Parent-Child Relationships" OR "Relationship, Parent-Child" OR "Mother-Child Relations"[Mesh] OR "Mother Child Relations" OR "Mother-Child Relation" OR "Relation, Mother-Child" OR "Relations, Mother-Child" OR "Mother-Child Interaction" OR "Interaction, Mother-Child" OR "Interactions, Mother-Child" OR "Mother Child Interaction" OR "Mother-Child Interactions" OR "Mother-Child Relationship" OR "Mother Child Relationship" OR "Mother-Child Relationships" OR "Relationship, Mother-Child" OR "Relationships, Mother-Child" OR "Mother-Infant Interaction" OR "Interaction, Mother-Infant" OR "Interactions, Mother-Infant" OR "Mother Infant Interaction" OR "Mother-Infant Interactions" OR "Mother-Infant Relations" OR "Mother Infant Relations" OR "Mother-Infant Relation" OR "Relation, Mother-Infant" OR "Relations, Mother-Infant" OR "Father-Child Relations"[Mesh] OR "Father Child Relations" OR "Father-Child Relation" OR "Relation, Father-Child" OR "Relations, Father-Child" OR "Father-Child Relationship" OR "Father Child Relationship" OR "Father-Child Relationships" OR "Relationship, Father-Child" OR "Relationships, Father-Child" OR "Child"[Mesh] OR "Children" OR "Child, Preschool"[Mesh] OR "Preschool Child" OR "Children, Preschool" OR "Preschool Children" OR "Infant" OR "Infants")) NOT (Animals)) NOT (Review [Publication Type])) AND ((randomized controlled trial[pt] OR controlled clinical trial[pt] OR clinical trials as topic[mesh:noexp] OR trial[ti] OR random*[tiab] OR placebo*[tiab])).
